# Impact of nutrition and rotavirus infection on the infant gut microbiota in a humanized pig model

**DOI:** 10.1186/s12876-018-0810-2

**Published:** 2018-06-22

**Authors:** Anand Kumar, Anastasia N. Vlasova, Loic Deblais, Huang-Chi Huang, Asela Wijeratne, Sukumar Kandasamy, David D. Fischer, Stephanie N. Langel, Francine Chimelo Paim, Moyasar A. Alhamo, Lulu Shao, Linda J. Saif, Gireesh Rajashekara

**Affiliations:** 1 Food Animal Research Program, The Ohio Agricultural Research and Development Center,Veterinary Preventive Medicine Department, The Ohio State University, 1680 Madison Avenue, Wooster, OH 44691 USA; 2 The Molecular and Cellular Imaging Center, The Ohio State University, Wooster, OH USA; 3 Present address: Group B-10: Biosecurity and Public Health, Bioscience Division, Los Alamos National Laboratory, Los Alamos, NM USA; 4 Present address: Hillman Cancer Center, University of Pittsburgh, 4200 Fifth Ave, Pittsburgh, PA 15260 USA

**Keywords:** Rotavirus, Humanized pig, Microbiota, Protein diet, Malnutrition

## Abstract

**Background:**

Human rotavirus (HRV) is a major cause of viral gastroenteritis in infants; particularly in developing countries where malnutrition is prevalent. Malnutrition perturbs the infant gut microbiota leading to sub-optimal functioning of the immune system and further predisposing infants to enteric infections. Therefore, we hypothesized that malnutrition exacerbates rotavirus disease severity in infants.

**Methods:**

In the present study, we used a neonatal germ free (GF) piglets transplanted with a two-month-old human infant’s fecal microbiota (HIFM) on protein deficient and sufficient diets. We report the effects of malnourishment on the HRV infection and the HIFM pig microbiota in feces, intestinal and systemic tissues, using MiSeq 16S gene sequencing (V4-V5 region).

**Results:**

Microbiota analysis indicated that the HIFM transplantation resulted in a microbial composition in pigs similar to that of the original infant feces. This model was then used to understand the interconnections between microbiota diversity, diet, and HRV infection. Post HRV infection, HIFM pigs on the deficient diet had lower body weights, developed more severe diarrhea and increased virus shedding compared to HIFM pigs on sufficient diet. However, HRV induced diarrhea and shedding was more pronounced in non-colonized GF pigs compared to HIFM pigs on either sufficient or deficient diet, suggesting that the microbiota alone moderated HRV infection. HRV infected pigs on sufficient diet showed increased microbiota diversity in intestinal tissues; whereas, greater diversity was observed in systemic tissues of HRV infected pigs fed with deficient diet.

**Conclusions:**

These results suggest that proper nourishment improves the microbiota quality in the intestines, alleviates HRV disease and lower probability of systemic translocation of potential opportunistic pathogens/pathobionts. In conclusion, our findings further support the role for microbiota and proper nutrition in limiting enteric diseases.

**Electronic supplementary material:**

The online version of this article (10.1186/s12876-018-0810-2) contains supplementary material, which is available to authorized users.

## Background

There is growing interest in understanding the effects of malnourishment in infancy and subsequent implications later in life [1–3]. Human breast milk is a nutritious complete food and it is considered as a ‘gold’ standard for infant nutrition [4, 5]. In conditions where breast-feeding is not possible or breast milk is not available in adequate quantities, infant formula provides an alternative safe and nutritious diet for infants [3]. In developing countries, deprivation of nutritious diet (infant formula or breast milk) due to various reasons (sanitation, infection, poverty etc) frequently leads to malnourishment of infants [6]. Malnutrition has devastating health consequences and increases the probability of contracting life-threatening diseases such as diarrhea, measles, pneumonia, malaria, and human immunodeficiency syndrome [7]. Malnutrition and enteric diseases form a vicious cycle because enteric diseases are more likely to occur in a malnourished host, and enteric pathogens aggravate malnutrition symptoms. This vicious cycle is difficult to overcome without proper intervention [8, 9]. Gastrointestinal infections affect the nutritional status due to mal-absorption of dietary intake, electrolyte imbalance, and secretory diarrhea, which lead to severe dehydration and malnourishment [8]. On the other hand, malnutrition results in intestinal dysbiosis, sub-optimal immune function, and increased gut permeability leading to a higher probability of translocation of opportunistic pathogenic bacteria or pathobionts and secondary infections [8, 9]. On either side of the vicious cycle ‘infection or malnutrition’, the gut microbiota acts as a bridge communicating responses and modulating the host metabolism [10]. The intestinal microbiota plays an important role in orchestrating host health. It supports host defense and homeostasis in recovery from enteric infections [11]. Abiotic or biotic stresses reduce the functionality of the microbiome and lower the production of metabolites usable by host [10]. It is now evident that the composition and activities of the gut microbiota drive various local and systemic effects [12]. Factors like xenobiotics (eg. probiotics, prebiotics or antibiotics) and enteric pathogens (eg human rotavirus, HRV) are also known to perturb the gut microbiota [12–14]. With the advent of next generation sequencing technology and the availability of bioinformatic tools, numerous studies have explored microbial ecology and the relevant microbiota functions in the host [12, 15–18]. For example, HRV infected infants displayed a reduction in the fecal microbiota diversity compared to healthy infants [19]. Thus, the role of the gut microbiota is increasingly recognized in health and disease.

HRV gastroenteritis is a vaccine preventable disease in infants that accounts for approximately 215,000 deaths annually worldwide [20]. The majority of these deaths occur in developing countries where malnourishment is common [20]. Although vaccines are available, their efficacy is low in developing countries [21, 22]. The poor efficacy of HRV vaccines in developing countries is attributed to numerous reasons including malnutrition and the dysbiotic gut microbiota [21, 23, 24]. Malnutrition perturbs the gut microbiota and thereby induces negative effects on the host immune system. Therefore, malnutrition is likely to contribute to HRV vaccine failure in developing countries [24, 25]. Identifying the specific microbial structure and composition in malnutrition and/or HRV infection has both diagnostic and therapeutic value, but not yet been fully investigated.

Due to various confounding factors and ethical concerns, addressing these questions in human infants is not possible. Human microbiota transplanted (microbiota humanized) animal models are used whereby selective microbial communities can be modeled under controlled conditions; however, not all microbiota humanized animal models recapitulate most of the donor microbiota (mouse microbiota humanized model) [26, 27]. Numerous publications have suggested pigs as a biologically relevant and non-primate model for transplanting human gut microbiota compared to rodent models [28–30]. Pigs are more advantageous non-primate models to study human conditions than rodents, because pigs are more closely related to humans in terms of anatomy, genetics, physiology and immunology and they are omnivores and outbred like humans [27, 31]. Transplantation of the human microbiota into germfree (GF) piglets resulted in comparable microbial community structure to the original specimen [26, 27, 32]. In contrast, humanizing GF mice with human microbiota did not recapitulate most of the microbial profiles seen in the original human donor stool [18, 33]. Therefore, GF piglets transplanted with human intestinal microbiota are increasingly recognized as a clinically relevant model to investigate the effects of diets and enteric pathogens on the intestinal microbiota [30, 34]. Importantly, GF pigs infected with HRV exhibit clinical signs and intestinal lesions similar to those seen in human infants, unlike the lack of HRV lesions and clinical disease in adult mouse models [27, 35]. We hypothesized that the transplantation of human infant fecal microbiota (HIFM) into GF pigs would result in a similar assembly and composition of microbiota in the gut and furthermore, malnutrition would alter the gut microbiota leading to sub-optimal functioning of the immune system, and exacerbating HRV disease severity.

In the present study, we transplanted GF pigs with HIFM and evaluated the impact of diet on gut microbiota composition and HRV disease susceptibility. Our results indicated that HIFM pigs on a malnourished diet displayed clinical symptoms mimicking the symptoms in malnourished infants and characterized by alteration of the gut microbiota and increased susceptibility to HRV disease.

## Methods

### Source of human infant fecal microbiota

Multiple fecal samples were aseptically collected in sterile fecal cups from a healthy, two-month-old, breast-fed, full-term male infant. Neither infant nor mother had any recent history of disease or antibiotic treatment at the time of sample collection. Fecal sample collection and use was approved by the Ohio State University Institutional Review Board protocol (protocol #2016H0276). Collected fecal samples were pooled and stored immediately at -80 °C until processed. Before freezing, a small aliquot of sample was tested for the presence of HRV using CCIF assay as described previously [36]. Fecal samples were weighed, diluted 1:20 (*w*/*v*) in phosphate buffer solution containing 0.05% cysteine (*v*/v) and 30% sterile glycerol as described previously [14]. Homogenized fecal suspensions were used to prepare 2 ml inoculum in an anaerobic working station (Microbiology International, MD) and were stored at -80 °C until inoculation.

### Transplantation of GF pigs with the HIFM

Near-term sows (Landrace × Yorkshire × Duroc cross-bred) were purchased from the Ohio State University Swine Center facility. Caesarean-derived GF piglets were housed individually in sterile positively pressured isolators (Alloy Fabricators Inc. Ohio, USA) to ensure no environmental contamination throughout the experiment [37]. The isolators were sterilized using the SPOR-KLENZ® Ready To Use kit (STERIS®, Ohio, USA) and their sterility was confirmed before and after housing of the GF piglets by aerobic and anaerobic cultures of environmental swabs of the isolators using blood agar. Further, the sterility of the GF piglets before HIFM transplantation was also confirmed by aerobic and anaerobic cultures of rectal swabs using blood agar. From derivation and during the course of experiment, piglets were maintained on either the sufficient diet or protein-calorie deficient diet. Piglets from a sufficient diet groups (groups 1, 3, and 5) were fed with bovine whole milk (Parmalat) containing 3.3% of protein and fat to provide adequate amounts of protein and fat, and 5% carbohydrate to maintain adequate amounts of calories despite lower fat content in cow vs. sow milk. Piglets from a deficient diet groups (groups 2, 4, and 6) were fed with Parmalat milk diluted in sterile water (1:1 ratio) [38]. By consequence, deficient diet groups were fed with a protein content twice lower than the recommended, mimicking a protein-energy deficient diet. Classical culture methods were used to confirm the sterility of GF piglets prior to HIFM transplantation as mentioned previously [39]. Further all GF piglets were confirmed negative for rotavirus, transmissible gastroenteritis virus, porcine epidemic diarrhea virus, calicivirus/sapovirus, astrovirus, and kobuvirus before transplantation [40–44]. Required numbers of HIFM inoculums were thawed prior to oral inoculation of GF piglets.

As a proof of concept, we performed oral inoculation of HIFM to GF piglets on sufficient diet. HIFM pigs were sacrificed at PTD7 to assess the microbiota composition in pig gut tissues and compared to the original HIFM sample (Additional file 1: Figure S1).

### Experimental design

All animal experiments were approved and performed in accordance to the Institutional Animal Care and Use Committee of The Ohio State University (Protocol #2010A00000088). The GF piglets (*n* = 24) were randomly divided into six groups (Fig. 2); Group-1: (sufficient diet HIFM+HRV; *n* = 5); Group 2 (deficient diet HIFM+HRV; *n* = 5); Group 3 (sufficient diet No HIFM+HRV; *n* = 5); Group 4 (deficient diet No HIFM+HRV; *n* = 3); Group 5 (sufficient diet HIFM+No HRV; *n* = 3) and Group 6 (deficient diet HIFM+No HRV; *n* = 3). Pigs in groups 1,2, 5, and 6 were transplanted once with 2 ml of original HIFM feces inoculum on day 4. Pigs in groups 1–4 were challenged with Wa(G1P [8]) HRV on day 14.

For microbial analysis, fecal samples were collected on PTD3, PTD6, PTD10/PCD0, PCD2, PCD5 and PCD12. At PCD14, all pigs were euthanized; small intestinal (duodenum, jejunum, ileum, and colon) and systemic tissues (spleen, liver and MLN) samples were collected aseptically in liquid nitrogen. All investigators involved in the sampling and testing were blinded to the animal group allocation. For long-term storage, samples were stored at -80 °C until processed for DNA extraction.

### Assessing clinical and pre-clinical correlates

Clinical signs, changes in body weight, HRV shedding, diarrhea severity and duration were recorded by trained animal technicians during the experiment. The severity of diarrhea was assessed based on the fecal consistency score [45]. Scores were recorded as: 0, normal; − 1, pasty; − 2, semiliquid; and − 3, liquid and pigs with daily fecal consistency scores of ≥1.5 were considered as diarrheic. The mean cumulative score was calculated as sum of daily fecal scores from each group from PCD0 to PCD7. HRV shedding in fecal samples was measured using a CCIF technique as described previously [36]. Cells were examined using an inverted fluorescence microscope and titers were expressed as FFU/ml.

### Genomic DNA extraction

- Fecal samples- fecal swabs collected from piglets were suspended in 2 ml sterile buffered peptone water. Suspensions were centrifuged at 10,000X g for 10 min and approximately 0.2 g sediment was used for genomic DNA extraction using PowerFecal DNA Isolation Kit (Mo Bio Laboratories, Carlsbad, CA) in accordance with the manufacture instruction. DNA was eluted from spin column using 100 μl of nuclease free water.
- Tissue sample- genomic DNA was extracted using DNeasy Blood and Tissue Kit (Qiagen, Valencia, CA). Briefly, approximately 0.25 g of tissue samples were cut into small pieces and suspended in buffer with proteinase K, and incubated at 56 °C for 3 h with intermittent vortexing. Subsequently samples were treated with RNase A (2 mg/ml) and ethanol precipitated. Suspension was transferred to spin column and washed. Finally, 200 μl of nuclease free water was used to elute DNA from the spin column. Quantity and quality of eluted DNA was assessed using NanoDrop 1000 Spectrophotometer V3.7.1 (Fisher Scientific, Pittsburgh, PA) and also by agarose gel electrophoresis.

### Amplicon library preparation and MiSeq sequencing

Extracted DNA samples were subjected for 16S rRNA V4-V5 variable region sequencing. As a first step of targeted sequencing, amplicon libraries were prepared by using Phusion® High-Fidelity PCR Kit (New England Biolabs Inc., Ipswich, MA) in a 96 well plate. Twenty five μl of PCR reactions were prepared using 5 μl (5X) of PCR buffer, 4 μl (5 ng/μl) of DNA sample, and 2.5 μl (2 μM) primer, 0.5 μl (10 mM) dNTPs, 0.2 μl of enzyme and finally nuclease free water was added to make-up the final volume. The barcoded primers targeted the region between V4-V5 variable region. Following PCR conditions were used for amplifications: initial denaturation was at 96 °C for 2 min, and 25 cycles of 96 °C for 30 s, 55 °C for 30 s, 72 °C for 30 s, with final extension of 72 °C for 5 min. Following PCR amplification PCR products were cleaned using AMPure XP PCR (Beckman Coulter Inc., Beverly MA). Samples’ concentrations were measured and equal concentration of all samples were pooled into one flow cell and sequenced using Illumina MiSeq 300-base, paired-end kit at the Molecular and Cellular Imaging Center located (https://mcic.osu.edu/genomics/illumina-sequencing).

### Bioinformatics analyses

The sequences were demultiplexed using bcl2fastq (v2.17; Illumina, Inc). In addition, samples that were pooled using in-line barcodes were demultiplexed using Sabre (https://github.com/najoshi/sabre). The resulting forward and reverse sequences were merged using Pandaseq (https://github.com/neufeld/pandaseq). During this step, any sequence with less than 0.7 threshold overlap was removed and primers used for amplification were trimmed. Controls containing only water and the extractions buffers used for the DNA extraction steps were also analyzed to confirm the lack of contaminants. Then samples were processed using Quantitative Insights Into Microbial Ecology (QIIME) software [46]. Operational Taxonomy Units (OTUs) were determined by clustering reads against Greengenes 16S reference dataset (2013–08 release) at 97% identity using open picking reference OTU (pick_open_reference_otus.py) method using default parameters, except setting minimum OTU size to 10. Microbial diversity was studied after rarefication of the sequences based on the lowest number of sequences among the samples tested. Alpha and beta diversities were analyzed using the core analysis package (core_diveristy_analyses.py), which included the comparison of the phylogenetic diversity and richness, principal coordinate analysis, and relative abundance studies (summarize_taxa_through_plots.py). Identification of microbial difference between different diets was performed using linear discriminant analysis (LDA) in the Galaxy / Hutlab website (https://huttenhower.sph.harvard.edu/galaxy/). Results were displayed via a plot cladrogram [47].

### Statistical analysis

Statistical analysis of the clinical and para-clinical correlates was done in GraphPad Prism 5 (GraphPad Software, Inc., CA, USA). Mean fecal HRV shedding, diarrhea scores, and normalized weight gain were compared by two-way ANOVA (ANOVA- general linear model), followed by Tukey’s multiple comparison test. *P* value of ≤0.05 was considered as significant. Analysis of the OTU relative abundance between treatments was analyzed in the Galaxy|Hutlab website using a linear discriminant analysis effective size (LefSe; http://huttenhower.sph.harvard.edu/galaxy/). A Kruskall-Wallis test combined with a pairwise Wilcoxon test was performed to identify statistical differences. P value of ≤0.01 was considered as significant.

## Results

### Transplantation of HIFM into GF pigs resulted in intestinal microbiota representative of the original specimen

**Fig. 1:**
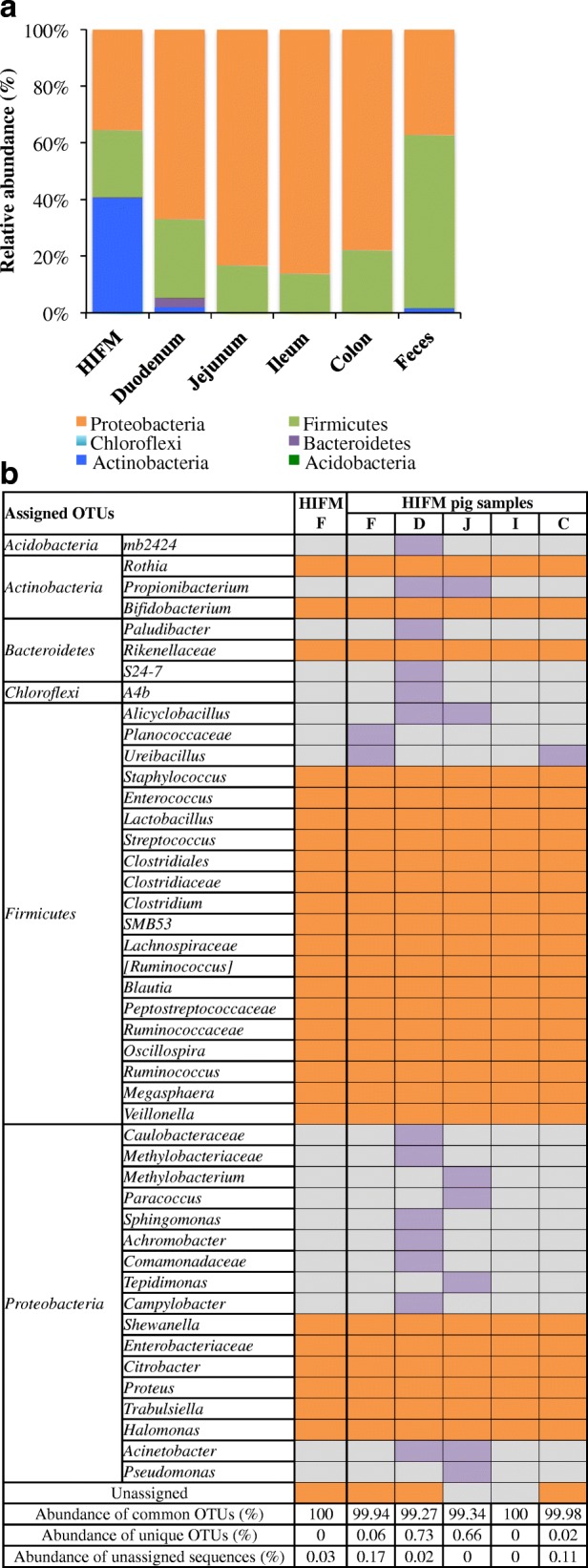
Microbiota data of HIFM transplantation into GF pigs at PTD7. **a** Microbiota relative abundance at the phylum level. **b** Microbiota comparison between the HIFM original sample and HIFM pig samples. In orange is the microbiota shared between original HIFM and HIFM pig feces and intestinal tissues; In purple are the unique OTUs detected only in HIFM pigs; and in grey are the OTUs undetected for the designated sample. HIFM and HIFM F: original HIFM feces; F: HIFM pigs feces; D: HIFM pigs duodenum; J: HIFM pigs jejunum; I: HIFM pigs ileum; C: HIFM pigs colon

Our goal was to use a GF pig model transplanted with HIFM to study the effects of malnutrition on the host microbiota and HRV infection. We transplanted the HIFM into four-days-old neonatal GF piglets on a protein sufficient diet (Additional file 1: Figure S1). Intestinal colonization was analyzed on post transplantation day (PTD) seven (Fig. 1). After preprocessing and taxonomic assignment with the Greengene database, a total of 308,752 sequences with a sequencing depth of 10,940 to 91,657 (mean = 51,458) reads per sample were analyzed in HIFM transplanted pig samples (*n* = 5). To study the beta diversity, each HIFM pig sample was normalized to 10,900 sequences, allowing the analysis of all samples.

Comparative analysis of the microbiota showed that the HIFM pig shared the majority of OTUs identified in the original HIFM sample but at different proportions. For example, at the phylum level *Actinobacteria*, *Proteobacteria* and *Firmicutes* were the most abundant with 40, 35, and 24%, respectively in the original HIFM sample, while *Proteobacteria* were the most abundant in the HIFM pig intestinal samples (between 67 to 86%) followed by *Firmicutes* (between 13 to 27%; Fig. 1a). In the HIFM pig fecal sample, *Firmicutes* were the most abundant with 61% followed by *Proteobacteria* with 37% (Fig. 1a). Furthermore, at the genus level, between 99.27 and 100% of the cumulative OTUs identified in the HIFM pig intestinal and fecal samples were represented in the original HIFM samples (Fig. 1b). These results confirm that at PTD7, the HIFM pig gut microbiota was stable and qualitatively representative of the original HIFM used. Based on these results, the piglet infection with HRV was performed at PTD10.

### Protein deficient diet resulted in decreased body weight gain in both the HIFM transplanted and non-transplanted pigs

**Fig. 2:**
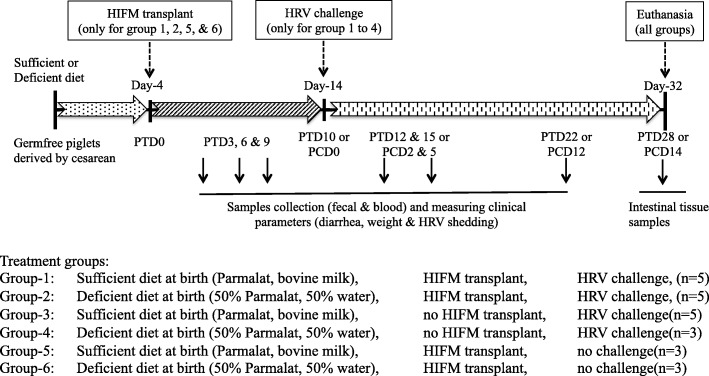
Schematics of animal experiment indicating times of HIFM transplantation, HRV challenge, and samples collection. Pigs were transplanted at 4 days of age, challenged at 14 days with 10^6^ FFU/pig of HRV Wa(G1P [8]) human strain, and euthanized at 32 days of age (dotted arrows). Tissues sampling and measurement of clinical parameters were indicated by solid arrows. HIFM-Human infant fecal microbiota; PTD-Post transplant days; PCD-Post HRV challenge days

**Fig. 3:**
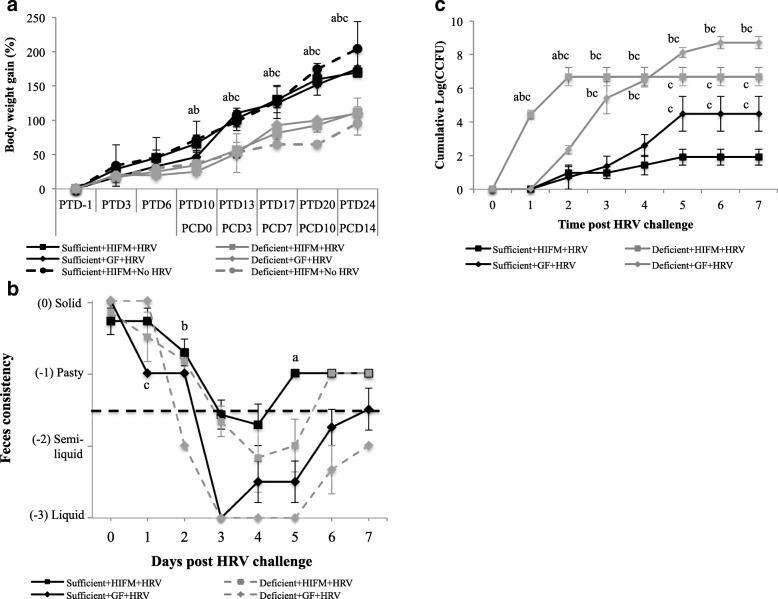
Impact of diet and the microbiota on body weight gain, diarrhea, and HRV shedding. **a** Body weight gain; a, b c, sufficient diet pig groups had significantly higher body weight gain than the deficient groups for HIFM+HRV challenged, GF+HRV, or HIFM+No HRV pigs respectively (*P* < 0.01). Bars represent standard errors. **b** Severity of diarrhea after HRV challenge. In black, pigs nourished with a sufficient diet; in grey, pigs nourished with a deficient diet. Diarrhea scoring was based on the phenotypic scale from 0 to − 3, where zero was for solid feces; − 1 when feces were pasty; − 2 when feces were semi-liquid; and − 3 when feces were liquid. -1.5 was the threshold where any values below were considered as typical diarrhea symptoms. a or b: deficient diet group had significantly lower diarrhea score than the sufficient diet group for the HIFM or GF pigs respectively; c: the sufficient diet HIFM group had significantly lower diarrhea score than the deficient diet HIFM group (*P* < 0.05). Bars represent standard errors. **c** HRV shedding. The letters a, b, or c indicate the cumulative log (CCFU) of the designated group significantly higher than the Deficient+GF+HRV, Sufficient+GF+HRV, or Sufficent+HIFM+HRV group at the corresponding time point, respectively (*P* < 0.05). Bars represent standard errors

Nutritional status and microbiota diversity are the two important factors contributing to host health, disease resistance, and body weight gain. These two parameters are even more important during the early infancy growth following birth [48]. Favorable growth conditions post-birth significantly enhance body weight gain and disease resistance over time. The goal of our experiment was to understand the importance of nutrition and microbial diversity and their resistance to HRV induced diarrhea. For these studies, GF pigs were given protein deficient or sufficient diets starting at birth and throughout the experiment, transplanted with or without HIFM, and in the presence or absence of HRV. An overview of the animal experimental design is depicted in Fig. 2. We determined the impact of HIFM, HRV infection, and diet on the body weight gain and results were displayed as body weight gain based on the initial weight recorded the day before HIFM transplantation (Fig. 3a). At the beginning of the experiment (PTD − 1), pig weight was approximately 2.87 ± 0.60 kg. Before HRV challenge (from PTD-1 to PTD6), none of the pig groups displayed significant differences in body weight gain; however sufficient diet pigs exhibited slightly higher weight gains compared to the deficient groups in both the HIFM and GF groups (*P* > 0.05). By the post-HRV challenge day zero (PCD0/PTD10), all three groups on sufficient diet (HIFM+HRV, GF+HRV and HIFM+No HRV) had a significant increase in the body weight compared to the deficient pig groups (*P* < 0.05). This difference between the sufficient and deficient groups became more pronounced, leading to a significant enhancement in body weight for the sufficient diet groups; HIFM+HRV (62.7%), GF+HRV (58.3%), HIFM+No HRV (108.9%) by the end of the experiment (PTD24/PCD14) compared to the deficient diet groups (*P* < 0.01). Further, HIFM in the pig gut did not significantly influence the capacity of the pigs to gain weight in both the sufficient and deficient diet groups (*P* > 0.05).

### Malnourished pigs exhibited severe diarrhea and enhanced virus shedding following HRV infection

Malnutrition affects the gut barrier which further increases the diarrheal symptoms in infants [9]; however, few studies have investigated HRV diarrheal severity with respect to malnutrition [49, 50]. To rule out the possibility of the dietary treatment or HIFM transplantation inducing diarrhea before HRV challenge, we monitored the diarrheal scores of pigs in different groups at three different time points (PTD 5, 7 and 9) prior to HRV challenge. Neither sufficient nor deficient diet induced any diarrhea (diarrhea score < 1.5) prior to HRV challenge in the HIFM and GF pigs. To determine the effect of the diet on HRV induced diarrhea and virus shedding, we compared diarrheal scores and fecal virus shedding between different groups for 7 days following HRV challenge (PCD0 to PCD7; Fig. 3b and c). On PCD5, HIFM+HRV pigs on deficient diet exhibited significant increase in diarrheal scores (*P* < 0.05) compared to HIFM+HRV pigs on sufficient diet (Fig. 3b). GF + HRV pigs on deficient diet also showed significant increase in diarrheal score at PCD2 compared to the sufficient GF+HRV group (*P* < 0.05). Overall, diarrhea was more severe in the GF + HRV groups and particularly in deficient pigs, suggesting that to some extent HIFM reduced HRV diarrhea severity.

Further, HRV shedding was quantified in feces using cell culture immunofluorescence (CCIF) assay [51, 52]. As in case with diarrhea, the diet and the microbiota affected HRV shedding (Fig. 3c). HIFM+HRV pigs on deficient diet started to shed HRV on PCD1, while the other groups started to shed the virus on PCD2. Differences in duration and titers of HRV shed were observed depending on the diet and presence or absence of HIFM. Although deficient diet HIFM+HRV group started shedding HRV earlier, no virus shedding was observed after PCD2; while, sufficient diet HIFM+HRV group continued to shed low titers of virus until PCD5. The GF+HRV pigs on a sufficient and deficient diets shed virus until PCD5 and PCD6, respectively. The GF+HRV groups had also higher titers of HRV and longer shedding compared with the HIFM+HRV groups. This was more pronounced in pigs on deficient diet. The deficient diet HIFM+HRV group had shorter shedding but shed higher titers of HRV compared with the sufficient diet HIFM+HRV group.

### Microbiota analysis in feces, intestine and systemic tissues of HIFM pigs

To identify interactions between the diet, microbial diversity, and HRV infection, we determined the microbiota composition in feces, intestinal tissues and systemic tissues. Fecal samples were collected before (PTD3, PTD6, PTD10 = PCD0) and after HRV challenge (PCD2, PCD5 and PCD12) from HIFM groups on either sufficient or deficient diet (Fig. 2). Similarly, intestinal samples (duodenum, jejunum, ileum, and colon) and other internal tissues (liver, MLN, spleen) were also analyzed to determine the impact of diet and HRV infection on gut microbiota composition and its systemic dissemination.

After preprocessing and taxonomic assignment with the Greengene reference database, 2,506,056 sequences were obtained for a total of 164 samples. Sequencing depth varied between 1030 and 73,881 reads per sample (mean_feces_ = 21,304; mean_intestines_ = 26,522; mean_systemic tissues_ = 4363). To study the microbiota abundance and diversity, HIFM pig samples were normalized to 1065 sequences for the fecal samples, 1150 sequences for the intestinal samples, and 1030 sequences for the systemic tissue samples.

Analysis of the alpha diversity displayed no significant differences in the phylogenetic diversity (Additional file 2: Figure S2 A, C, and E) and richness (Additional file 2: Figure S2 B, D, and F) when feces, intestine, systemic tissue samples from HIFM+HRV and HIFM+No HRV groups were analyzed based on the diet only and ignoring the time points, intestinal location, or systemic tissues. The deficient pig feces and tissue samples consistently displayed a slightly higher alpha diversity and richness than the sufficient samples. No distinct spatial separation or clustering of the feces, intestine, or systemic tissue samples were detected based on the diet when the principal coordinate analysis (PCoA) were performed (Additional file 2: Figure S2 G, I, and K); on the other hand, the presence of HRV seemed to induce slight shifting of the microbiota in intestines and systemic organs of HIFM+HRV samples compared to the HIFM+No HRV samples (Additional file 2: Figure S2 J and L).

### HRV infection altered fecal microbiota diversity and abundance

**Fig. 4:**
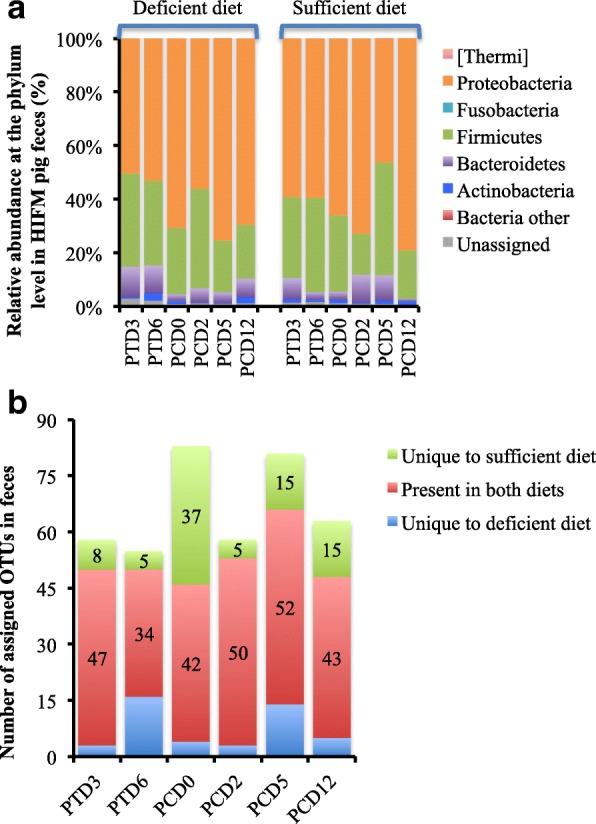
Beta diversity analysis of fecal samples before and after HRV challenge from HIFM pigs on a deficient or sufficient diets. **a** Relative abundance at the phylum level. **b** Microbiota diversity over time between sufficient and deficient diets based on the OTU assignment after open OTU picking with the Greengene database. PTD- Post HIFM transplant day; PCD- Post HRV challenge day

Detailed analysis of the beta diversity displayed variations in the relative abundance between sufficient and deficient fecal samples from HIFM pigs at the phylum level over time (Fig. 4a). Before challenge, phyla abundance displayed low differences between diets (sufficient and deficient) and time points (PTD3, PTD6, and PCD0). *Proteobacteria* and *Firmicutes* were the most abundant phyla, representing together more than 90% of the relative abundance, followed by *Bacteroidetes*. More variations were detected after HRV challenge, mostly in *Firmicutes*, *Bacteroidetes*, and *Proteobacteria*. A decrease in *Firmicutes* was observed at PCD2 in sufficient diet HIFM+HRV pig feces (15 ± 22%) compared with the deficient diet HIFM+HRV pig feces (37 ± 6%) and there was an increase in *Proteobacteria* abundance in sufficient diet HIFM+HRV pig feces (73 ± 11%) compared with the deficient diet HIFM+HRV pig feces (56 ± 20%). The opposite trend was observed at PCD5 and PCD12. *Firmicutes* were increased in sufficient diet HIFM+HRV pig feces at PCD5 and PCD12 (42 ± 7% and 34 ±4% respectively) compared to deficient diet HIFM+HRV pig fecal samples (19 ± 28% and 20 ±19%, respectively). These trends were not observed with the other phyla; however, *Bacteroidetes* increased in sufficient diet HIFM+HRV pig feces by at least two-fold at PCD2 and PCD5 compared to deficient diet HIFM+HRV pig feces. Despite distinct differences in *Firmicutes* and *Bacteroidetes* abundances observed between the sufficient and deficient diets HIFM groups in the feces (Fig. 4a), no associations were detected when the *Firmicutes*:*Bacteroidetes* ratios were compared to the body weight gain data (Fig. 3a). The sufficient and deficient diets HIFM groups displayed similar ratios between PTD3 and PCD2. The deficient HIFM+HRV pigs had higher ratios at PCD5 (3.9 ± 0.5) compared to the sufficient HIFM+HRV pigs (1.1 ± 0.1). It is only at PCD12 a higher ratio in sufficient HIFM+HRV pigs (18.8 ± 4.9) compared to the deficient ones (2.4 ± 0.7) was observed; however, this trend was not consistent thus making these results not conclusive.

Alterations of the microbiome diversity were observed within fecal samples over time (Fig. 4b). Overall, fecal samples from sufficient diet HIFM pigs had a slightly higher number of unique assigned OTUs (in green) than deficient ones (in blue). The only exception was at PTD6, where OTU numbers were higher in deficient diet HIFM pig feces.

**Fig. 5:**
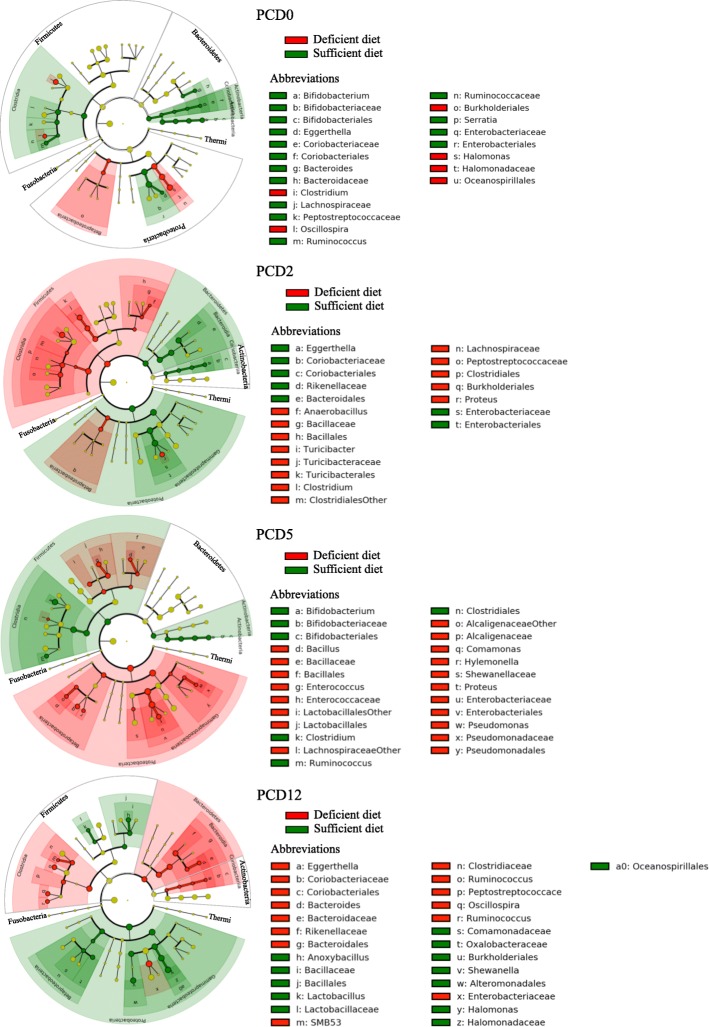
Impact of the diets on fecal microbiota of HIFM+HRV pigs. The relative abundance between deficient and sufficient diets for a given time point is shown. Results are represented via a phylogenetic tree (Graphlan), combined with relative abundance data. Labeled in red are the bacteria detected in higher abundance in deficient diet; while in green are the bacteria detected in higher abundance in sufficient diet. The labeling of the taxonomic levels from the outside (phylum) to the inside (genus), while the tree start (root) from the center and goes outside. Nodes are indicated by a circle. Bacteria (node) more abundant in one of the diets is shown in red or green, no change is shown in gold. Bacteria are designated with alphabet in red or green corresponding to the node

The global comparison of fecal microbiota between diets before and after HRV infection is shown in Additional file 3: Figure S3 and Fig. 5. Disparities in the microbiota were observed between diets following HIFM transplantation (Additional file 3: Figure S3) and HRV challenge (Fig. 5); however the differences were not significant.

### A sufficient diet combined with HRV infection increased the microbiota diversity in the gut

**Fig. 6:**
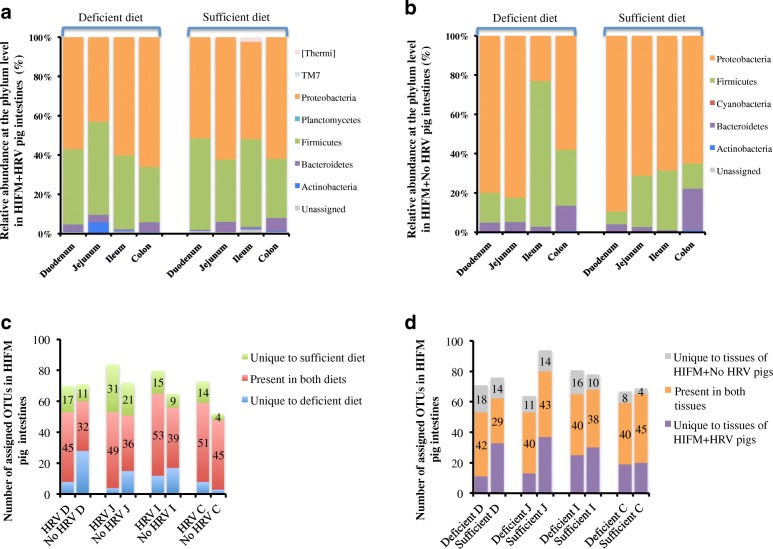
Beta diversity analysis of intestinal samples from HIFM+HRV and HIFM+No HRV pigs nourished with deficient or sufficient diet. Comparison of relative abundances at the phylum level between diets for the HIFM+HRV (**a**) and HIFM+No HRV (**b**) pigs. Microbiota diversity in intestinal tissue samples based on diet (**c**) or HRV challenge status (**d**). OTUs were assigned by open OTU picking with the Greengene database. D: Duodenum; J: Jejunum; I: Ileum; C: Colon; HRV: HRV challenged tissue; Non-HRV: Non-HRV challenged tissue

Analysis of beta diversity showed that bacterial populations at the phylum level were similar between intestinal locations and also between diets for HRV challenged samples at PCD14 (Fig. 6a). *Proteobacteria* and *Firmicutes* were the most abundant phyla, representing together more than 90% of the relative abundance identified in the tissues, followed by *Bacteroidetes*. *Bacteroidetes* were higher in deficient diet HIFM+HRV pig duodenum while *Actinobacteria* were higher in deficient diet HIFM+HRV pig jejunum. Same analysis with the non-HRV challenged samples showed variations between intestinal locations and also between diets (Fig. 6b). The comparison between deficient and sufficient groups for each intestinal location displayed pronounced increase in *Firmicutes* abundance in ileum of sufficient diet HIFM+No HRV pigs (74 ± 38%) compared to deficient diet HIFM+No HRV (30 ± 27%) and HIFM+HRV (40 ± 26%) groups. The increase in *Firmicutes*; coincided with reduction in *Proteobacteria* abundance in ileum of sufficient diet HIFM+No HRV pigs (23 ± 24%) compared to deficient diet HIFM+No HRV (68 ± 39%) and + HIFM+HRV (53 ± 23%) groups. The general comparison of the HRV challenged with the non-challenged samples showed that *Firmicutes* were more abundant in the HRV challenged intestinal tissues; while *Proteobacteria* were more abundant in the non-HRV challenged intestinal tissues (Fig. 6a and b).

Furthermore, distinct modifications of the microbiome composition were observed in the intestinal tract based on the diet and HRV challenge status (Fig. 6c and d). In most cases, HIFM+HRV groups had a higher diversity at the OTUs level than the HIFM+No HRV groups. The only exception was with the duodenum of the deficient diet HIFM pigs. Also, the intestine of HIFM+HRV pigs on a sufficient diet (in green) consistently had a higher number of unique OTUs than the intestine of HIFM+HRV pigs on a deficient diet (in blue). Some specific OTUs seemed to be selectively affected depending on HRV challenge status. For example, intestine of HIFM+HRV groups were more diverse in *Bacillales*, *Lactobacillus*, *Caulobacterales*, and *Thermales* compared to the HIFM+No HRV groups. Moreover, the diversity of these groups of bacteria was also influenced by the diets and the intestinal locations, suggesting that HRV infection and nutrient availability in different intestinal locations may induce specific selection pressures on the microbiota.

**Fig. 7:**
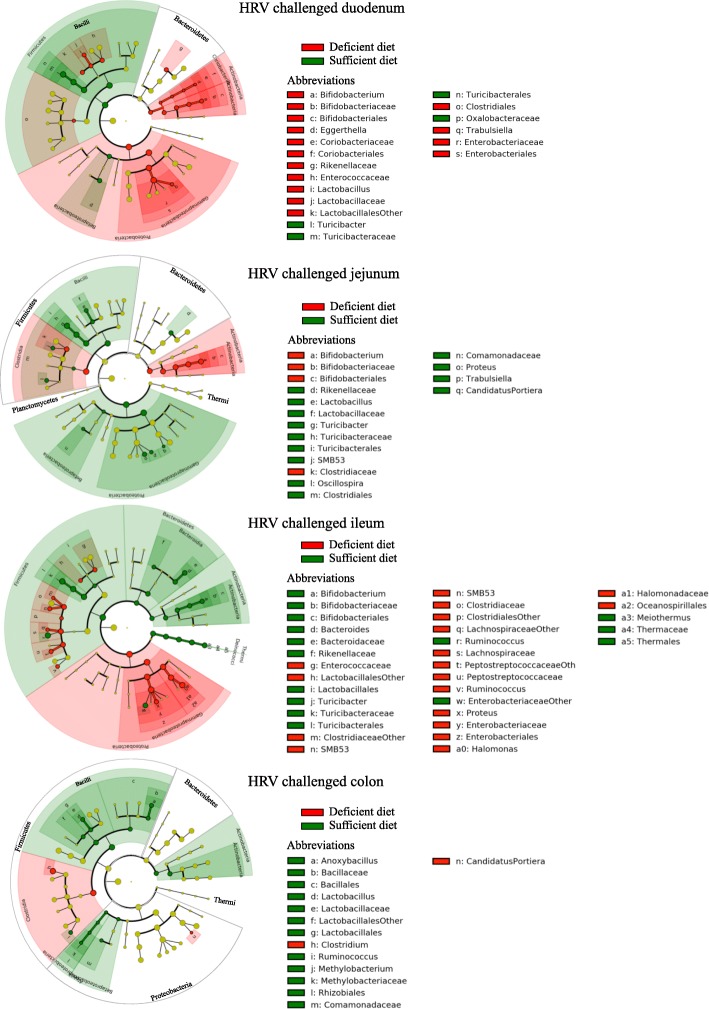
Impact of the diets on the intestinal microbiota in HIFM+HRV pigs. The relative abundance between deficient and sufficient diets for a given tissue is shown. Results are represented via a phylogenetic tree (Graphlan), combined with relative abundance data. Labeled in red are the bacteria detected in higher abundance in deficient diet; while in green are the bacteria detected in higher abundance in sufficient diet. The labeling of the taxonomic levels from the outside (phylum) to the inside (genus), while the tree start (root) from the center and goes outside. Nodes are indicated by a circle. Bacteria (node) more abundant in one of the diets is shown in red or green, no change is shown in gold. Bacteria are designated with alphabet in red or green corresponding to the node

These observations were supported by a plot cladrogram combined with the relative abundance data (Fig. 7), which showed the effect of diet on the predominance of certain bacterial populations in the intestinal samples. For example, *Firmicutes* were always more abundant in the intestine of HIFM+HRV pigs on a sufficient diet with *Bacilli* such as *Turicibacteraceae*, while *Firmicutes* were more abundant in the intestine of HIFM+HRV pigs on a deficient diet with *Clostridia*. A deficient diet seemed to increase the *Proteobacteria* abundance, mainly with the *Gammaproteobacteria* (*Enterobacteriaceae*), while a sufficient diet seemed to increase the *Proteobacteria* abundance mainly the *Betaproteobacteria*. *Actinobacteria* were more abundant in the upper intestinal tract of HIFM+HRV pigs on a deficient diet, while they were more abundant in the lower intestinal tract of HIFM+HRV pigs on a sufficient diet. However, in the non-challenged intestinal tissues, no trends were detected (Additional file 4: Figure S4).

### An increased microbial dissemination was observed in systemic tissues in HRV infected deficient pigs

**Fig. 8:**
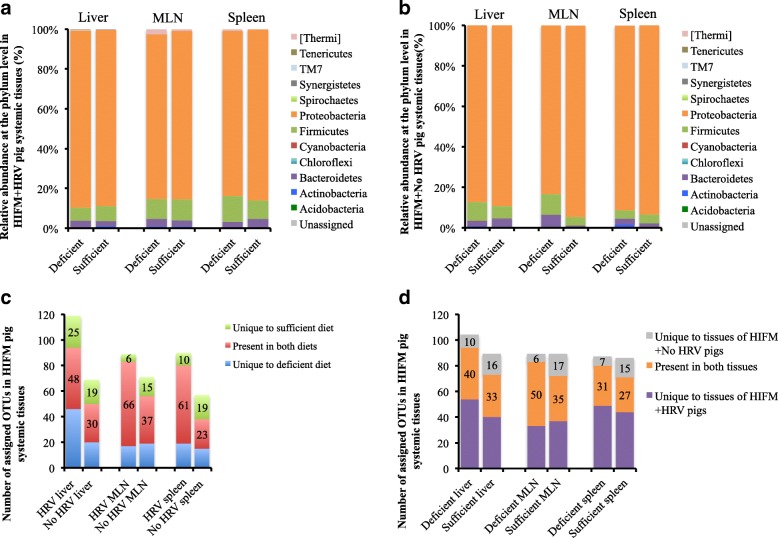
Beta diversity analysis of systemic tissues samples from HIFM+HRV and HIFM+No HRV pigs nourished with a deficient or sufficient diet. Comparison of relative abundances at the phylum level between diets for the HIFM+HRV (**a**) and HIFM+No HRV (**b**) pigs. Microbiota diversity in systemic tissues samples based on diet (**c**) or HRV challenge status (**d**). OTUs were assigned by open OTU picking with the Greengene database. HRV: HRV challenged tissue; Non-HRV: Non-HRV challenged tissue

Analysis of the beta diversity displayed very similar bacterial populations at the phylum level between systemic tissues, diets, and HRV challenge status (Fig. 8a and b). *Proteobacteria* was the most abundant phylum with more than 90% relative abundance, followed by the *Firmicutes* and *Bacteroidetes*. *Proteobacteria* was also the most abundant phylum in HIFM+HRV pig tissues; however, these samples displayed more fluctuations in the microbiota abundance based on the diet and showed disparities in the HRV challenged tissues. For example, in MLN *Proteobacteria* were more abundant in the sufficient diet HIFM+No HRV pigs (94 ± 1%) compared to sufficient diet HIFM+HRV pigs (84 ± 10%) and the deficient diet HIFM+No HRV pigs (83 ± 14%); this increase of *Proteobacteria* in MLN was also coincided with a decrease in *Firmicutes* and *Bacteroidetes* for the sufficient diet HIFM+No HRV pigs. Spleen tissues of HIFM+No HRV pigs also had less *Firmicutes* (~ 4%) than the spleen tissues from HIFM+HRV pigs (~ 11%) in both diet groups.

Distinct modifications of the microbiota diversity were observed in the tissues depending on the diet and HRV infection (Fig. 8c and d). HIFM+HRV pig tissues displayed a higher total number of OTUs compared to HIFM+No HRV pig tissues, suggesting that HRV infection increased the dissemination of enteric bacteria in these tissues. Also, tissues of HIFM+HRV pigs on a deficient diet (in blue) always had a higher number of OTUs than tissues of HIFM+HRV pigs on a sufficient diet (in green), suggesting that the diet also influenced the microbial dissemination to systemic tissues. Liver and MLN had slightly higher OTUs in the deficient diet HIFM+No HRV group compared with the sufficient diet HIFM+No HRV group; however, an opposite trend was observed in the spleen.

**Fig. 9:**
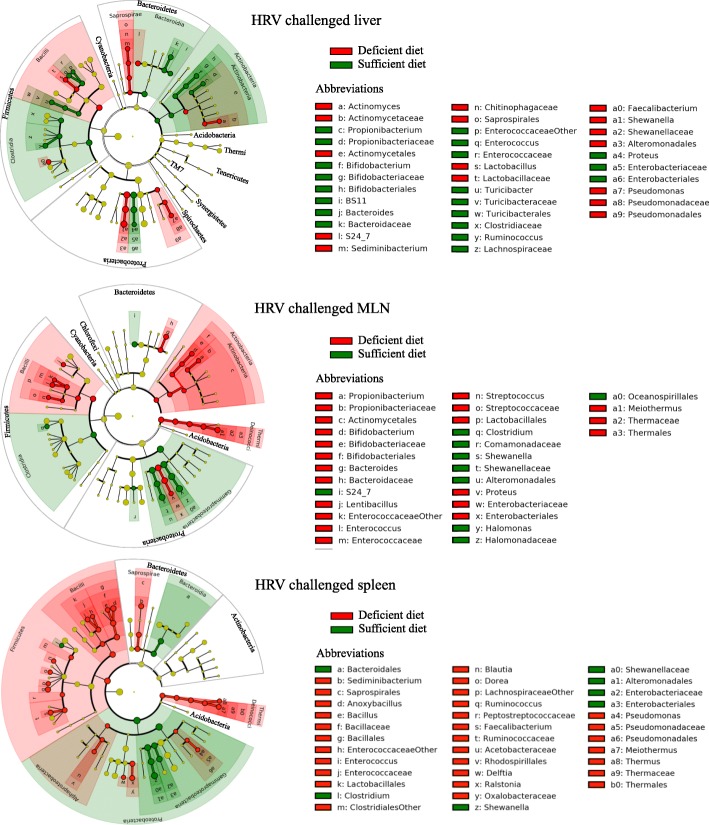
Impact of the diets on the systemic tissues microbiota of HIFM+HRV pigs. The relative abundance between deficient and sufficient diets for a given tissues is shown. Results are represented via a phylogenetic tree (Graphlan), combined with relative abundance data. Labeled in red are the bacteria detected in higher abundance in deficient diet; while in green are the bacteria detected in higher abundance in sufficient diet. The labeling of the taxonomic levels from the outside (phylum) to the inside (genus), while the tree start (root) from the center and goes outside. Nodes are indicated by a circle. Bacteria (node) more abundant in one of the diets is shown in red or green, no change is shown in gold. Bacteria are designated with alphabet in red or green corresponding to the node

Figure 9 and Additional file 5: Figure S5 show the diet effect on the predominance of certain bacterial taxa for each systemic tissue. For example, *Thermi* were always higher in tissues of deficient diet HIFM+HRV pigs compared with the sufficient diet HIFM+HRV group, while this trend was inconsistent in HIFM+No HRV pig tissues. Unlike the intestinal tissues (Fig. 7), sufficient diet increased the abundance of *Clostridia*, while deficient diet increased the abundance of *Bacilli* for both liver and MLN of HIFM+HRV pigs. In the spleen of HIFM+HRV pigs, deficient diet induced a general increase of the *Firmicutes* in both *Clostridia* and *Bacilli* classes.

## Discussion

Rotavirus accounts for up to 40% of infant diarrheal deaths [53] and combined with an imbalanced nutrition, rotavirus is one of primary causes of mortality and morbidity worldwide [54]. An infectious dose as low as 10 virulent HRV particles is sufficient to infect and cause diarrhea in a susceptible individual. Once a child is infected, he can spread the infection up to 50% of the children in close contact, increasing HRV incidence [55–58]. Hence, the amount of HRV shedding and the duration of shedding in infected individuals are of paramount importance in HRV diarrheal outbreaks. Nutritional status and gut-microbiota play significant roles in maintaining gut barrier function [9, 59, 60]. Perturbations of these two parameters have additive effects on the persistence of malnutrition and enteric infections [9, 61]. The triad of ‘diet-gut microbiota-host response’ is important in an individual’s overall development but more importantly in infants due to the recent concept of ‘the first 1000 days of life’ [62].

Although, not many studies have investigated the impact of diet, HRV infection, and gut microbiota in humans, only a few studies (including ours) have tried to mimic the human infant microbiome in animal models, using neonatal GF piglet transplanted with HIFM to study these parameters [50]. Our pilot study showed that at PTD7 more than 99% of the bacterial diversity present in the original HIFM fecal samples from a two-month-old baby was represented all along the pig intestines and in feces. Different proportions of bacteria were detected in the tissues studied, suggesting that some bacteria grow better in pigs depending on the intestinal location. For example, *Bifidobacterium* was present in higher abundance in the original HIFM fecal samples and 10 times less in the HIFM pig intestinal and fecal samples. The original HIFM sample was obtained from a breast fed baby, while HIFM pigs were formula fed. *Bifidobacterium* are frequently transferred from mother-to-infant, and it has been shown that breast-feeding increases the diversity and abundance of *Bifidobacteria* [63–65]. As expected, some bacteria not detected in the original HIFM fecal sample were detected in the HIFM transplanted pigs. However, these bacteria were lower than 0.7% in each pig tissue relative to the whole microbiota. It is likely that these unique bacteria were at very low concentration in the original HIFM fecal sample and were not detectable after sample processing for metagenomics studies. The diet may have contributed to the enrichment of these bacteria in pig gut. This was supported by the presence of unique bacteria mostly in the upper part of the intestine and less in the lower part. Despite these variations in the microbial population, our results suggested that 7 days are sufficient to have a representative colonization of the pig intestines by the original HIFM.

HRV infected malnourished piglets had significant reduction in body weight gain and an enhanced diarrhea [50]. A recent study also showed that malnutrition was significantly associated with more severe HRV induced diarrhea in infants [66]. We also demonstrated that sufficient diet facilitates more rapid recovery from diarrhea and increase body weight gain in piglets, highlighting the significance of nutritional strategies to moderate HRV infections. On the other hand, the gut microbial diversity did not affect the body weight of HRV challenged pigs, but the HIFM transplantation did significantly decreased the diarrhea severity and duration in both diet groups compared to the GF groups. Zijlstra et al., 1997 and Jacobi et al., 2013, also showed that the quality of the microbiome is an important factor in limiting HRV infection [38, 50]. These results suggest that the diet might affect the microbiome and host physiology, resulting in alterations in HRV infection and period of morbidity. For example, Zijlstra et al. showed that the decline in the body weight gain and severe diarrhea observed with malnourished piglets challenged with HRV were accompanied by a reduction in villus height and lactase activity, reduced villus:crypt height ratio, reduction in trans-epithelial resistance, and increase in intestinal insulin-like growth factor binding proteins (IGFBP) [38, 50].

HRV infection in infants was associated with decrease in the gut microbial diversity [19, 67]; however, in our study, an opposite trend was observed after analysis of intestinal tissues from HIFM+HRV pigs fed with either a sufficient or deficient diet compared to the HIFM+No HRV groups. This finding can be explained by the destruction of the intestinal cells by HRV, which could make more nutrients available for the microbes in the gut [68]. Furthermore, most of the infants’ studies rely on analysis of fecal samples collected from either mid or late phase of HRV infections [19, 67]. We also observed that the microbiota quality, not the abundance, in intestinal tissues of the HIFM+HRV pigs was different between the sufficient and deficient diets, suggesting that both HRV infection and the diet may have profound effect on microbial diversity and abundance. As a consequence, the modifications in microbial community caused by the diet could explain in part the reductions observed in clinical signs and bacterial translocation to systemic organs. Both deficient and sufficient diet HIFM+HRV groups displayed unique bacteria present only in one of the diet groups which could serve as biomarkers of HRV infection and may aid in development of novel strategies to moderate HRV diarrhea. For example, *Turicibacter,* and *Anoxybacillus* were detected only in HIFM+HRV pig intestines. Also, *Turicibacter, Halomonas,* and *Shewanella* were more abundant in the sufficient diet HIFM+HRV group, suggesting these bacteria could serve as potential bio-indicators of HRV infection and/or host nutrition. Previous association of *Turicibacter* species in colon and small intestine of mice was shown to possess immune-modulatory effects through T cells (CD8+) and NK cells activity [69]. Thus, it is likely that the presence of *Turicibacter* species in sufficient HIFM pigs may indicate modulation of immune response promoting recovery from HRV severity.

Unlike the microbiota in intestinal tissues, neither HRV infection nor the diet induced major modifications of the microbiota abundance in the systemic tissues; however, in concordance with impaired intestinal integrity [50], all systemic tissues of HIFM+HRV groups had a higher microbial diversity compared to the HIFM+No HRV groups, suggesting that HRV infection was associated with a general increase of the microbiota diversity in systemic tissues. Further the diet had an additive effect; however, the increase in diversity was enhanced when pigs were fed deficient diet. These results suggested that HRV infection increases the bacterial translocation to liver, MLN, and spleen likely by compromising the intestinal epithelial barrier; while malnutrition enhances this phenomenon by exacerbating intestinal damage caused by HRV infection [50].

Though our results clearly demonstrate the interconnections between the diet, microbiota and HRV infection, it should be taken into consideration that only limited number of pigs was used in each treatment group in this study due to the complex nature of experiments with the GF animals. The changes in the gut microbiota in our study may be due to individual or combined effects of the following factors: (i) malnutrition, as malnutrition was shown to affect gut microbiota structure and composition; (ii) HRV pathogenesis- previous studies have shown that enteropathogens including HRV have significant effects on the gut microbiota [14]; and (iii) the host response or immune response- the host natural defense system are essential for maintaining the homeostasis of the gut microbiota [62]. Recurrent episodes of diarrhea caused by enteropathogens have a major effect on the gut microbiota [9]. To substantiate this claim, previous studies have shown that malnourished children, who did not have a diarrheal disease, likely due to enteric infections, did indeed gain weight normally compared to well-nourished children, while the increasing incidence of recurrent diarrhea episodes in malnourished children progressively decreased the weight gain [70, 71]. Hence, in natural settings, it is clear that the recurrent episodes of diarrhea have the greatest effect on children’s growth likely due to their cumulative effects on gut microbiota with prolonged dysbiosis and intestinal absorptive dysfunction, which is especially problematic in undernourished children [9].

## Conclusions

In the present study, we showed that HRV infected malnourished HIFM piglets had perturbed gut microbiota and recapitulated the clinical signs read as seen in malnourished HRV infected infants. Our results showed that malnutrition superimposed with HRV infection increases the bacterial translocation to systemic organs further supporting the findings that malnutrition exacerbates HRV infection by compromising the intestinal epithelial barrier. Irrespective of the diet, presence of gut microbiome itself offers a certain degree of protection to HRV, as the GF pigs without resident gut microbiota displayed more severe form of disease. Further studies looking at the contribution of microbiota from malnourished infants from African countries, where malnutrition and HRV infection are highly prevalent, would provide greater insights into contribution of microbiota to the vicious cycle of ‘infection or malnutrition’. Though our study used 16S rRNA based approach to profile microbiota in malnutrition and HRV infection; metagenomic analysis using shot gun sequencing is needed to identify microbial consortium that can be manipulated to minimize HRV infection.

## Additional files

Additional file 1: **Figure S1.** Schematics of animal experiment indicating time of HIFM transplantation and time points of samples collection. Pigs were transplanted at 4 days of age and euthanized at 11 days of age (dotted arrows). Intestinal tissues sampling was performed at PTD7. Abbreviations: HIFM-Human infant fecal microbiota; PTD-Post transplant days. (PDF 36 kb)

Additional file 2: **Figure S2.** Comparison of the microbiota alpha diversity of HIFM pig samples based on the diets (deficient or sufficient) and HRV status (pre- and post- HRV challenge). Diversity in the feces (A & B), intestines (C & D), and systemic tissues (E & F) samples are shown irrespective of time points, intestinal locations, or tissues type. Alpha diversity was analyzed based on the rarefaction curve of the phylogenetic diversity and Choa1: richness. Bars represent the standard deviations. No significant differences were detected between diets for either the phylogenetic diversity or richness (*P* > 0.05). General composition of the HIFM pig samples microbiota using a principal coordinated analysis (PCoA). Samples were clustered based on the diet or HRV status for the feces (G & H), intestines (I & J), and systemic organs (K & L). (ZIP 362 kb)

Additional file 3: **Figure S3.** Impact of the diet on fecal microbiota of HIFM pigs before HRV challenge. The relative abundance between deficient and sufficient diets for a given time point is shown. Results are represented via a phylogenetic tree (Graphlan), combined with relative abundance data. Labeled in red are the bacteria detected in higher abundance in deficient diet; while in green are the bacteria detected in higher abundance in sufficient diet. The labeling of the taxonomic levels from the outside (phylum) to the inside (genus), while the tree start (root) from the center and goes outside. Nodes are indicated by a circle. Bacteria (node) more abundant in one of the diets is shown in red or green, no change is shown in gold. Bacteria are designated with alphabet in red or green corresponding to the node. (PDF 2487 kb)

Additional file 4: **Figure S4.** Impact of the diet on intestinal microbiota of HIFM+No HRV pigs. The relative abundance between deficient and sufficient diets for a given tissues is shown. Results are represented via a phylogenetic tree (Graphlan), combined with relative abundance data. Labeled in red are the bacteria detected in higher abundance in deficient diet; while in green are the bacteria detected in higher abundance in sufficient diet. The labeling of the taxonomic levels from the outside (phylum) to the inside (genus), while the tree start (root) from the center and goes outside. Nodes are indicated by a circle. Bacteria (node) more abundant in one of the diets is shown in red or green, no change is shown in gold. Bacteria are designated with alphabet in red or green corresponding to the node. (PDF 3708 kb)

Additional file 5: **Figure S5.** Impact of the diet on systemic tissue microbiota of HIFM+No HRV pigs. The relative abundance between deficient and sufficient diets for a given tissues is shown. Results are represented via a phylogenetic tree (Graphlan), combined with relative abundance data. Labeled in red are the bacteria detected in higher abundance in deficient diet; while in green are the bacteria detected in higher abundance in sufficient diet. The labeling of the taxonomic levels from the outside (phylum) to the inside (genus), while the tree start (root) from the center and goes outside. Nodes are indicated by a circle. Bacteria (node) more abundant in one of the diets is shown in red or green, no change is shown in gold. Bacteria are designated with alphabet in red or green corresponding to the node. (PDF 1746 kb)

## Abbreviations

ANOVA: Analysis of variance
CCIF: Cell culture immunofluorescence
DNA: Deoxyribonucleic acid
FFU: Focus forming unit
GF: Germ free
HIFM pig: Human infant fecal microbiota transplanted GF pigs
HIFM: Human infant fecal microbiota
HRV: Human rotavirus
LDA: Linear discriminant analysis
MLN: Mesenteric lymph nodes
OTU: Operational taxonomic unit
PCD: Post challenge day
PCoA: Principal coordinate analysis
PCR: Polymerase chain reaction
PTD: Post transplantation day
QIIME: Quantitative insights into microbial ecology
RNA: Ribonucleic acid
*v*/v: Volume per volume
*w*/*v*: Weight per volume
