# Supplementary figures and images for: Impact of nutrition and rotavirus infection on the infant gut microbiota in a humanized pig model
Source: BMC Gastroenterol. 2018 Jun 22;18:93. doi: 10.1186/s12876-018-0810-2 (PMC6013989; doi:10.1186/s12876-018-0810-2)

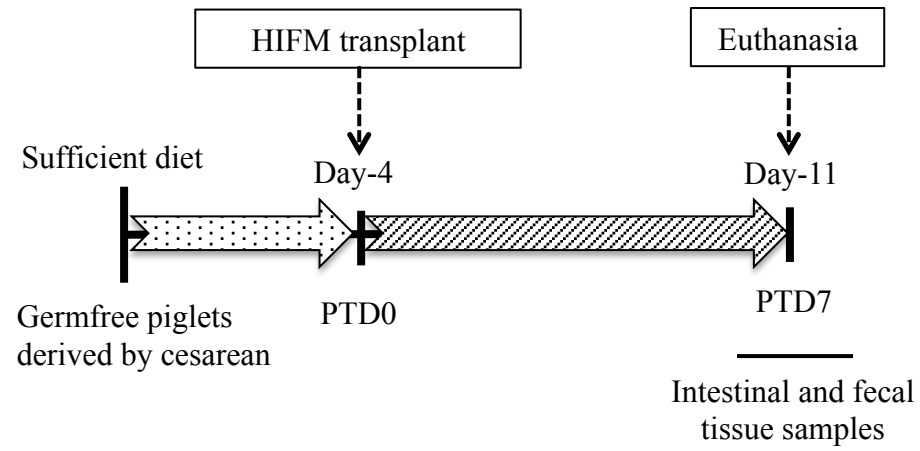

Supplement: Supplementary file 1 — Figure S1. Schematics of animal experiment indicating time of HIFM transplantation and time points of samples collection. Pigs were transplanted at 4 days of age and euthanized at 11 days of age (dotted arrows). Intestinal tissues sampling was performed at PTD7. Abbreviations: HIFM-Human infant fecal microbiota; PTD-Post transplant days. (PDF 36 kb) [file 12876_2018_810_MOESM1_ESM.pdf]

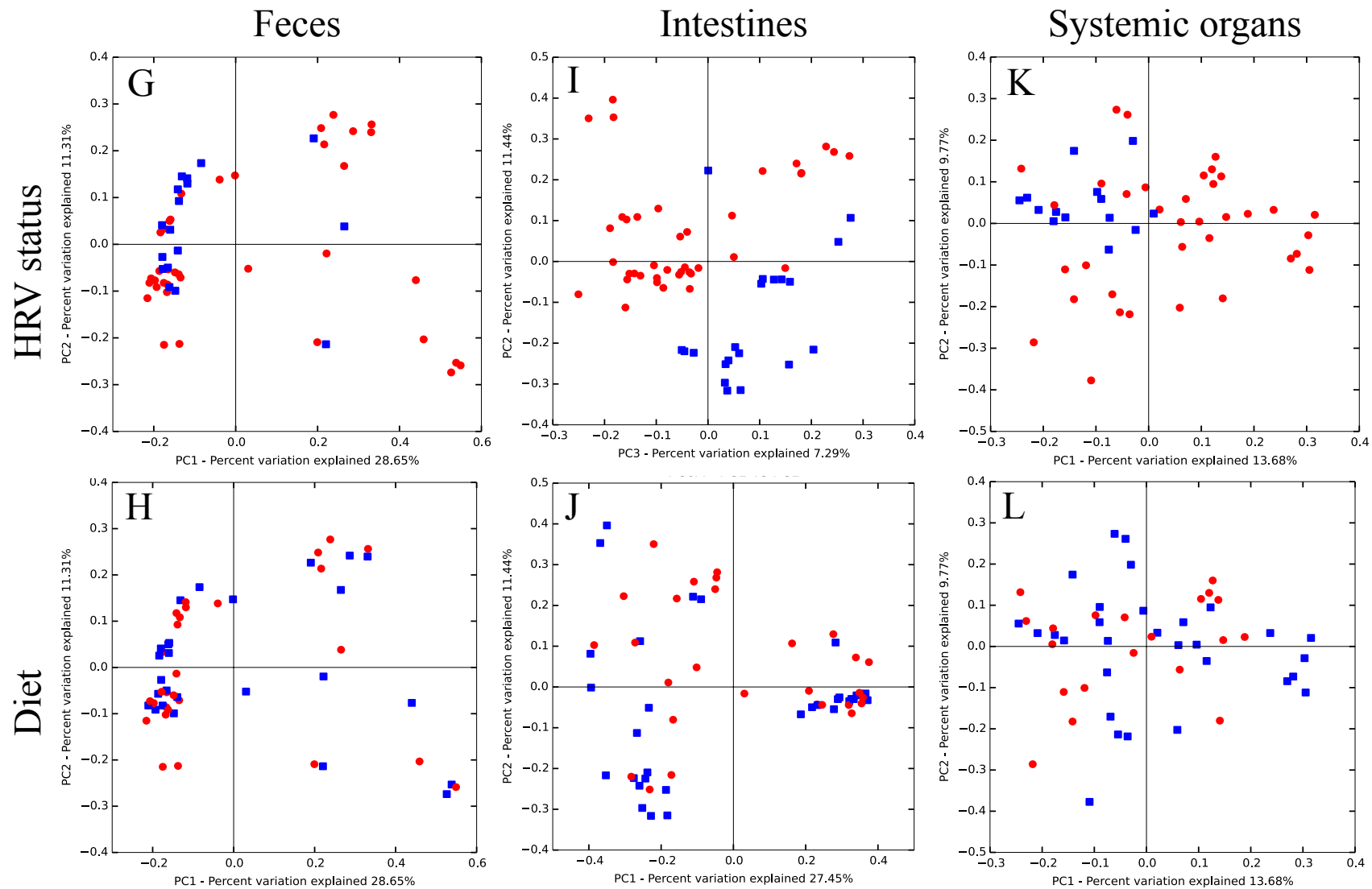

Supplement: Supplementary file 2 — Figure S2. Comparison of the microbiota alpha diversity of HIFM pig samples based on the diets (deficient or sufficient) and HRV status (pre- and post- HRV challenge). Diversity in the feces (A & B), intestines (C & D), and systemic tissues (E & F) samples are shown irrespective of time points, intestinal locations, or tissues type. Alpha diversity was analyzed based on the rarefaction curve of the phylogenetic diversity and Choa1: richness. Bars represent the standard deviations. No significant differences were detected between diets for either the phylogenetic diversity or richness (P > 0.05). General composition of the HIFM pig samples microbiota using a principal coordinated analysis (PCoA). Samples were clustered based on the diet or HRV status for the feces (G & H), intestines (I & J), and systemic organs (K & L). (ZIP 362 kb) [file 12876_2018_810_MOESM2_ESM.zip › Figure S2.2R4.pdf]

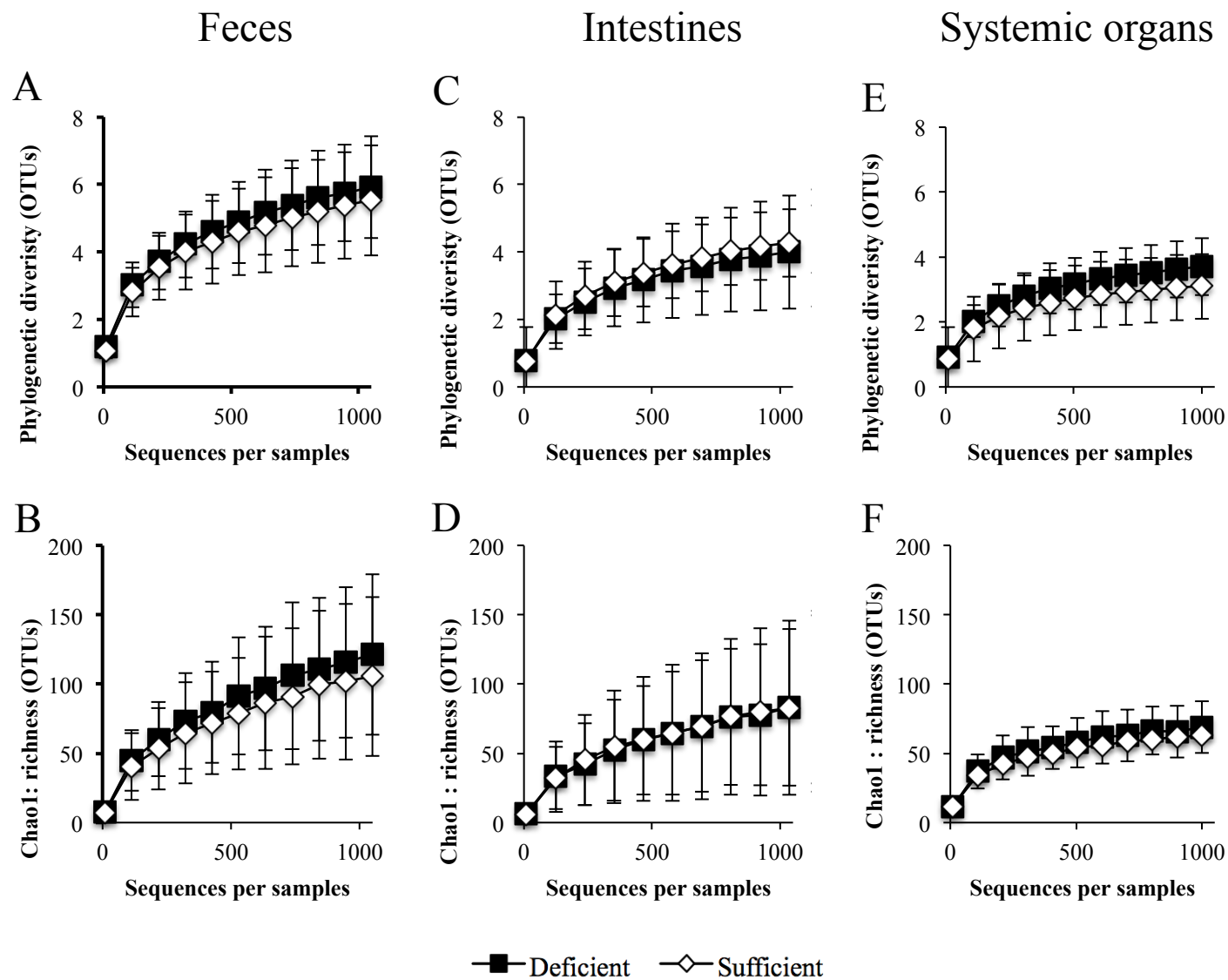

Supplement: Supplementary file 2 — Figure S2. Comparison of the microbiota alpha diversity of HIFM pig samples based on the diets (deficient or sufficient) and HRV status (pre- and post- HRV challenge). Diversity in the feces (A & B), intestines (C & D), and systemic tissues (E & F) samples are shown irrespective of time points, intestinal locations, or tissues type. Alpha diversity was analyzed based on the rarefaction curve of the phylogenetic diversity and Choa1: richness. Bars represent the standard deviations. No significant differences were detected between diets for either the phylogenetic diversity or richness (P > 0.05). General composition of the HIFM pig samples microbiota using a principal coordinated analysis (PCoA). Samples were clustered based on the diet or HRV status for the feces (G & H), intestines (I & J), and systemic organs (K & L). (ZIP 362 kb) [file 12876_2018_810_MOESM2_ESM.zip › Figure S2.1R4.pdf]
