# Supplementary material for: Impact of nutrition and rotavirus infection on the infant gut microbiota in a humanized pig model
Source: BMC Gastroenterol. 2018 Jun 22;18:93. doi: 10.1186/s12876-018-0810-2 (PMC6013989; doi:10.1186/s12876-018-0810-2)
Supplement: Supplementary file 4 — Figure S4. Impact of the diet on intestinal microbiota of HIFM+No HRV pigs. The relative abundance between deficient and sufficient diets for a given tissues is shown. Results are represented via a phylogenetic tree (Graphlan), combined with relative abundance data. Labeled in red are the bacteria detected in higher abundance in deficient diet; while in green are the bacteria detected in higher abundance in sufficient diet. The labeling of the taxonomic levels from the outside (phylum) to the inside (genus), while the tree start (root) from the center and goes outside. Nodes are indicated by a circle. Bacteria (node) more abundant in one of the diets is shown in red or green, no change is shown in gold. Bacteria are designated with alphabet in red or green corresponding to the node. (PDF 3708 kb) [file 12876_2018_810_MOESM4_ESM.pdf]

Non-HRV challenged duodenum

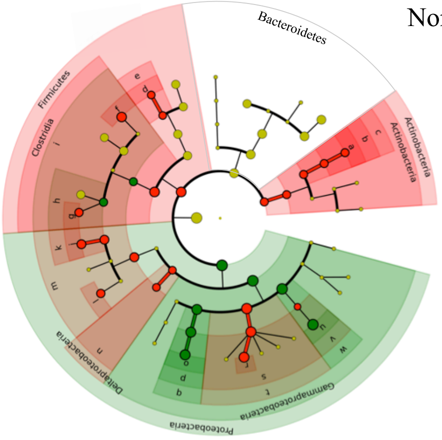

- Deficient diet
- Sufficient diet

Abbreviations

- a: Bifidobacterium
- b: Bifidobacteriaceae
- c: Bifidobacteriales
- d: Lactobacillus
- e: Lactobacillaceae
- f: Clostridium
- g: Oscillospira
- h: Ruminococcaceae
- i: Clostridiales
- j: Burkholderia
- k: Burkholderiaceae
- l: Ralstonia
- m: Burkholderiales
- n: Myxococcales
- o: Shewanella
- p: Shewanellaceae
- q: Alteromonadales
- r: EnterobacteriaceaeOther
- s: Enterobacteriaceae
- t: Enterobacteriales
- u: Halomonas
- v: Halomonadaceae
- w: Oceanospirillales

Non-HRV challenged jejunum

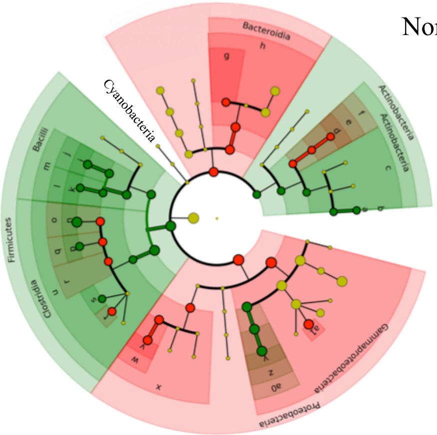

- Deficient diet
- Sufficient diet

Abbreviations

- a: Corynebacterium
- b: Corynebacteriaceae
- c: Actinomycetales
- d: Bifidobacterium
- e: Bifidobacteriaceae
- f: Bifidobacteriales
- g: S24_7
- h: Bacteroidales
- i: Enterococcus
- j: Enterococcaceae
- k: Lactobacillus
- l: Lactobacillaceae
- m: Lactobacillales
- n: Clostridium
- o: Clostridiaceae
- p: Ruminococcus
- q: Lachnospiraceae
- r: Peptostreptococcaceae
- s: Faecalibacterium
- t: Oscillospira
- u: Clostridiales
- v: Burkholderia
- w: Burkholderiaceae
- x: Burkholderiales
- y: Shewanella
- z: Shewanellaceae
- a0: Alteromonadales
- a1: EnterobacteriaceaeOther

Non-HRV challenged ileum

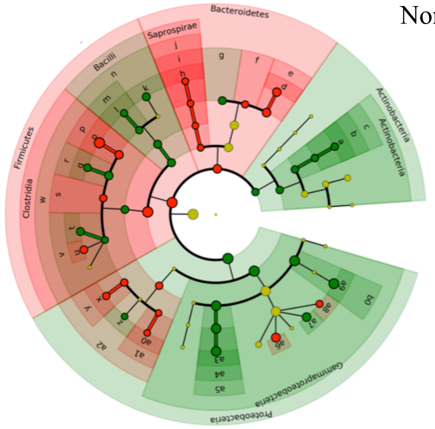

- Deficient diet
- Sufficient diet

Abbreviations

- a: Bifidobacterium
- b: Bifidobacteriaceae
- c: Bifidobacteriales
- d: Bacteroides
- e: Bacteroidaceae
- f: Rikenellaceae
- g: S24_7
- h: Sediminibacterium
- i: Chitinophagaceae
- j: Saprospirales
- k: Enterococcus
- l: Lactobacillus
- m: Lactobacillaceae
- n: Lactobacillales
- o: Clostridium
- p: Clostridiaceae
- q: Ruminococcus
- r: Lachnospiraceae
- s: Peptostreptococcaceae
- t: Faecalibacterium
- u: Oscillospira
- v: Ruminococcaceae
- w: Clostridiales
- x: Burkholderia
- y: Burkholderiaceae
- z: Delftia
- a0: Ralstonia
- a1: Oxalobacteraceae
- a2: Burkholderiales
- a3: Shewanella
- a4: Shewanellaceae
- a5: Alteromonadales
- a6: EnterobacteriaceaeOther
- a7: Proteus
- a8: Trabulsiella
- a9: Halomonas
- b0: Oceanospirillales

Non-HRV challenged colon

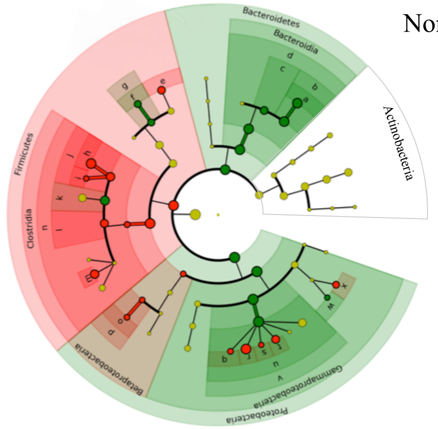

- Deficient diet
- Sufficient diet

Abbreviations

- a: Bacteroides
- b: Bacteroidaceae
- c: Rikenellaceae
- d: Bacteroidales
- e: Enterococcus
- f: Lactobacillus
- g: Lactobacillaceae
- h: Clostridium
- i: SMB53
- j: Clostridiaceae
- k: Lachnospiraceae
- l: Peptostreptococcaceae
- m: Oscillospira
- n: Clostridiales
- o: Burkholderia
- p: Burkholderiaceae
- q: Citrobacter
- r: EnterobacteriaceaeOther
- s: Pantoea
- t: Proteus
- u: Enterobacteriaceae
- v: Enterobacteriales
- w: CandidatusPortiera
- x: Halomonas
