# Supplementary material for: Impact of nutrition and rotavirus infection on the infant gut microbiota in a humanized pig model
Source: BMC Gastroenterol. 2018 Jun 22;18:93. doi: 10.1186/s12876-018-0810-2 (PMC6013989; doi:10.1186/s12876-018-0810-2)
Supplement: Supplementary file 5 — Figure S5. Impact of the diet on systemic tissue microbiota of HIFM+No HRV pigs. The relative abundance between deficient and sufficient diets for a given tissues is shown. Results are represented via a phylogenetic tree (Graphlan), combined with relative abundance data. Labeled in red are the bacteria detected in higher abundance in deficient diet; while in green are the bacteria detected in higher abundance in sufficient diet. The labeling of the taxonomic levels from the outside (phylum) to the inside (genus), while the tree start (root) from the center and goes outside. Nodes are indicated by a circle. Bacteria (node) more abundant in one of the diets is shown in red or green, no change is shown in gold. Bacteria are designated with alphabet in red or green corresponding to the node. (PDF 1746 kb) [file 12876_2018_810_MOESM5_ESM.pdf]

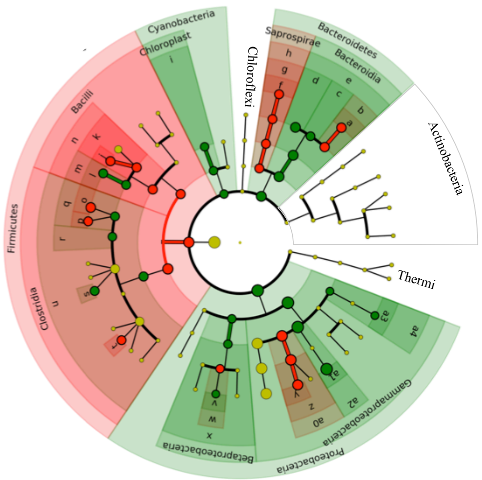


## Non-HRV challenged liver

- Deficient diet
- Sufficient diet

### Abbreviations

a: Bacteroides
b: Bacteroidaceae
c: Rikenellaceae
d: S24_7
e: Bacteroidales
f: Sediminibacterium
g: Chitinophagaceae
h: Saprospirales
i: Streptophyta
j: Enterococcus
k: Enterococcaceae
l: Lactobacillus
m: Lactobacillaceae
n: Lactobacillales
o: ClostridiaceaeOther
p: Clostridium
q: Clostridiaceae
r: ClostridialesOther
s: Ruminococcus
t: Faecalibacterium
u: Clostridiales
v: Delftia
w: Comamonadaceae
x: Burkholderiales
y: EnterobacteriaceaeOther
z: Enterobacteriaceae
a0: Enterobacteriales
a1: Halomonas
a2: Oceanospirillales
a3: Stenotrophomonas
a4: Xanthomonadales

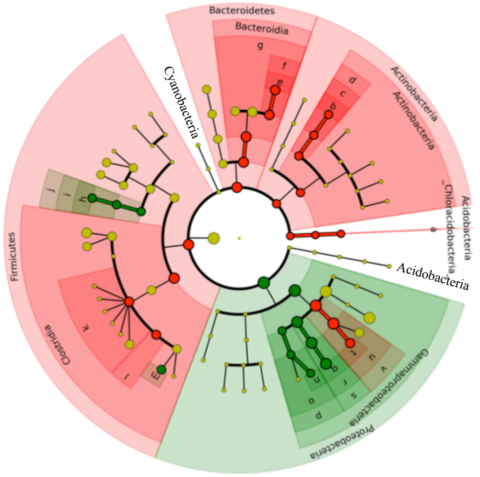


## Non-HRV challenged MLN

- Deficient diet
- Sufficient diet

### Abbreviations

a: RB41
b: Bifidobacterium
c: Bifidobacteriaceae
d: Bifidobacteriales
e: Bacteroides
f: Bacteroidaceae
g: Bacteroidales
h: Turicibacter
i: Turicibacteraceae
j: Turicibacterales
k: Lachnospiraceae
l: Peptostreptococcaceae
m: Faecalibacterium
n: Succinivibrio
o: Succinivibrionaceae
p: Aeromonadales
q: Shewanella
r: Shewanellaceae
s: Alteromonadales
t: Enterobacteriaceaeother
u: Enterobacteriaceae
v: Enterobacteriales
